# A focus on delocalization error poisoning the density-functional many-body expansion

**DOI:** 10.1039/d5sc90053k

**Published:** 2025-02-26

**Authors:** Barbaro Zulueta, John A. Keith

**Affiliations:** a Department of Petroleum and Chemical Engineering, University of Pittsburgh Pittsburgh PA 008815 USA jakeith@pitt.edu

## Abstract

Broderick and Herbert's article (D. R. Broderick and J. M. Herbert, *Chem. Sci.*, 2024, **15**, 19893–19906, https://doi.org/10.1039/D4SC05955G) explores an open concern about using energies from density-functional approximations when developing force fields and machine learning potentials for large-scale simulations. The authors explicitly decomposed self-interaction errors (SIEs) from density-functional approximations (DFAs) and found how they behave in many-body expansions (MBEs) that are leveraged in large-scale simulations. For DFAs to be deemed reliable for developing many-body potentials, they would ideally provide stable energetics within the MBE terms that are most often used by force fields and machine learning potentials (MLPs), *i.e.*, within their three- and four-body terms. It was instead found that many widely used DFAs produce wild oscillations in these MBE terms, whereby three-body terms can become problematically enormous. This raises concerns that any force field and/or MLP that appears well-fitted to DFA data on small systems might be poorly conditioned for large-scale simulations due to intrinsic SIEs. This commentary provides more context of Broderick and Herbert's work and its consequences for members of the multiscale modeling community.

## Abstract 2

Many-body expansions of quantum chemistry data are used for multiscale modeling, but density functional approximations with less than 50% of exact Hartree–Fock exchange can bring significant errors (Broderick *et al.*, *Chem. Sci.*, 2024, https://doi.org/10.1039/D4SC05955G).

## Graphical abstract

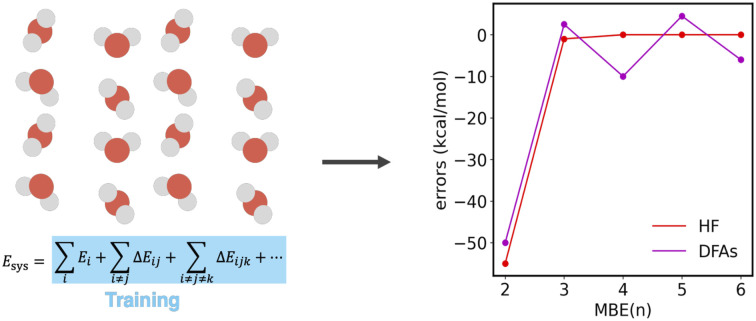

Kohn–Sham density functional theory (KS-DFT)^1,2^ is the most widely used quantum chemistry (QC) method due to its favorable balance of computational cost, accuracy, and transferability in determining electronic energies across chemical compound space. While wavefunction theory (WFT) methods map electronic energies with exact but computationally costly approximations based on orbital populations, KS-DFT maps ground-state electronic energies with less exact expressions based on electronic densities that bring less computational cost. No KS-DFT method has yet provided a physically exact exchange–correlation (XC) contribution, but density-functional approximations (DFAs), *e.g.*, the generalized-gradient approximation and hybrid functionals, employ parameterized schemes to recover as much XC as possible, and inexactness can still cause physical problems when modeling several important classes of systems.

For example, all DFAs carry self-interaction errors (SIEs) that arise in inexact treatments of exchange energies, and these errors cause modeled electronic densities to always be at least slightly more delocalized than they should be. Such errors will be negligible in electronically conductive systems, but in systems where electronic structures are more intricate, *e.g.*, cases of material semiconductor band gaps and molecular applications involving ions, radicals, vertical excitations, heteroatomic bond dissociation, and barrier heights, delocalization errors can cause catastrophically incorrect predictions.^3^ Hybrid (and double-hybrid) functionals are a transferable protocol to correct SIE errors, but these methods only mitigate SIEs to an imprecise extent and they are substantially more computationally expensive than non-hybrid DFAs, and therefore usually less desirable.

Despite these pernicious errors, DFAs continue to be used in the multiscale modeling community to train force fields and machine learning potentials (MLPs) that map system energies from DFAs onto computationally efficient many-body approximations that are better suited for large-scale simulations. Data-driven machine learning methods have been introduced to compute water clusters' many-body expansions (MBEs) from density-corrected DFT data.^4^ DFA descriptors on machine-learned many-body force fields have been used to model bulk solid and liquid phase interactions of d-block elements and ligand exchange in metal complexes.^5,6^ Others have developed simulation methods using environmentally dependent analytical many-body effects that require DFA descriptors (*e.g.*, densities, partial charges, and dipole moment) using either tight-binding^7^ or machine learning models.^8–10^ These works all have significant value to the community, but it is also possible that intrinsic and surreptitious errors from the DFAs might carry over into larger-scale simulations in problematic ways.

The impact of SIEs on their many-body energy expansions had not been previously studied explicitly, but Broderick and Herbert's work (https://doi.org/10.1039/D4SC05955G)^11^ provides a thorough investigation of intermolecular MBEs using KS-DFT to analyze fundamental interactions of water and aqueous ions (*i.e.*, X(H_2_O)_*n*_, where X is F^−^, Na^+^, and H_2_O and *n* is the number of H_2_O *n*-mers) *via* explicit/implicit solvation modeling. The authors employed their MBE fragmentation method^12^ with popular DFAs (*i.e.*, PBE, SCAN, SCAN0, PBE0, LRC-PBE, B3LYP, BH&H-LYP, HF-LYP, and ωB97X-V) and Hartree–Fock (HF) methods, all with various well-known mitigation/error cancellation strategies (*i.e.*, HF density-corrected DFT, counter-poise corrections, dielectric continuum boundary conditions, and energy-based screening) and different basis set sizes (*i.e.*, augmented double, triple, and quadruple zetas, and 6-31G).

The reported results may not surprise some, but they warrant broad attention. All of these DFAs, except for functionals greater than or equal to 50% exchange (*i.e.*, BH&H-LYP and HF-LYP), suffer from significant oscillations in their MBEs, even with higher-order energy expansions. Past work has shown that unphysical predictions due to SIEs can be mitigated with a sufficient amount of exact exchange,^13–15^ but this carries over into MBEs as well. Broderick and Herbert showed that the inherent delocalizing nature produced by SIEs was the leading cause for these size-extensive errors in the MBEs, especially when anions are present and bonding between the solute and solvent are dictated more by electrostatics in the form of hydrogen bonding than donor–acceptor bonds. The net effect is that MBEs based on DFAs will also contain SIEs, and these errors will get inherited into force fields and MLPs in ways that may become very problematic for large-scale simulations. Higher-order many-body corrections might correct these accumulating errors, but that brings inefficiencies from a simulation perspective.

Although counterintuitive to some in the community, Broderick and Herbert show that HF theory provides a better alternative for MBEs than most DFAs (except for functionals greater than 50% exchange) because its MBE errors are smaller for up to three bodies, and the MBE contributions monotonically decrease as *n*-body contributions increase. Unlike DFAs, the magnitude of the higher-order terms beyond the third order were negligible for HF, in part because HF does not suffer from SIEs. This means MBEs can be efficiently and satisfactorily constructed with up to three-body contributions, similar to dispersion models that usually contain two- and three-bodies.^16^ Furthermore, because HF is often the first step of any accurate WFT method (*e.g.*, CCSD(T) and MP*n* calculations), the authors recommended a cost-efficient approach that employs their many-body expansions on HF (with screening to consider only sufficiently close interactions) and then applying accurate post-HF methods that are free from SIEs to obtain accurate energetics for intermolecular interactions.

In conclusion, Broderick and Herbert show that SIEs present in DFAs create wild oscillations in their MBEs, and these errors become greater as the system size increases. This means that force field and machine learning potentials that employ MBEs and are trained to DFA descriptors may contain significant errors that would be deleterious for their predictive reliability, especially in larger simulations. Looking forward, we suspect that there will be more interest in developing diverse and multiscalable QC methods that are explicitly based on HF or other methods that are free from SIEs, *e.g.*, ref. 17–19. In tandem with MBE tools and procedures developed in Herbert's group, such efforts should be easier to implement.

## Author contributions

B. Z. and J. A. K. wrote the manuscript.

## Conflicts of interest

There are no conflicts to declare.
